# Some Recent Innovations

**Published:** 1910-01-01

**Authors:** 


### Anæsthetics

### SOME RECENT INNOVATIONS.

Of new anæsthetics, new methods of administering old ones, even new principles altogether, the present century, especially during the last three or four years, has been very prolific. There are in addition a good many of the old problems still unsolved, or still matters of dispute.

### Scopolamine-Morphine.

Of this combination, injected subcutaneously before the patient is brought to the operating-theatre, much has lately been written. In Scotland, America, and on the Continent many anæsthetists speak most highly of it; but somehow it has not gained much acceptance with English administrators, and several who have tried it seem to regard it as distinctly unsafe. This conflict of opinion is somewhat perplexing, in view of the enthusiasm displayed across the border and across the Atlantic (see The Hospital, November 21, 1908, p. 187, and October 23, 1909, p. 94). The latest paper on the use of this combination appears in the December issue of the *Edinburgh Medical Journal*, by Drs. H. T. Thomson and D. Cotterill. Two methods have been employed by these authors: one is the usual plan of giving $\frac{1}{6}$ gr. of morphine with 1/64 gr. scopolamine hydrobromate two hours before the operation; the other is a recommendation of Dr. Bertha van Husen, who gives $\frac{1}{8}$ gr. morphine with 1/120 gr. scopolamine two and a half hours before, another similar dose an hour later, and a third dose half an hour before operation. The latter plan produces results which differ only in degree from those of the former, the effect of divided dosage being the more pronounced. It has often been found possible to give the third and even the second injection without waking the patient; and if chloroform be selected for the induction stage it is also possible to get that over without the patient becoming conscious of the administration. Many patients, for instance, have no recollection afterwards of having been removed from the ward.

The conclusions of these anæsthetists are summed up thus. As the result of either the single or the repeated dose, fear as a source of danger is avoided. They lay much stress on this point, and quite rightly: there is little doubt that the mysterious early syncope in some fatal cases of anæsthetic misadventure may be due partly or mainly to the terror of the highly-strung and nervous patient. The amount of anæsthetic required is small, and in many cases none is needed after the first skin incision. For most abdominal sections it will be necessary, in order to obtain sufficient relaxation to suit the surgeon, to maintain light general anæsthesia throughout. When a few whiffs of anæsthetic only are given for the commencement of the operation, there is no trouble with shock, even in such serious operations as amputation of the arm or leg. The mucous and salivary secretions are inhibited by the drugs, and therefore troubles on this score are avoided. As regards post-anæsthetic vomiting the results are good: it is not altogether abolished, but is, on the average, markedly reduced. Lastly, the patients sleep for some hours after the operation, and are thus spared a great deal of pain and discomfort. It is a suggestion worth bearing in mind that scopolamine-morphine would be a useful preliminary to spinal analgesia, and would overcome largely the disadvantages which sometimes result from the acute perceptions of the patient to what is going on around him. Bier, amongst others, has already tried this plan. (*Deutsche Zeitschrift für Chirurgie, Bd.* xcv., p. 373.)

### Face Burns.

From time to time cases occur, though not many of them are reported, of severe burns of the face, and of acute conjunctivitis, caused during anæsthesia by contact of the liquid anæsthetic with the skin or conjunctiva. Every textbook mentions this possibility, and advises the use of vaseline as a prophylactic measure. As a matter of fact, few anæsthetists do actually smear their patient's faces (and their own fingers) with this emollient; and in practice it is quite easy to avoid any such sources of annoyance to the patient. Elementary care in the handling of the drop-bottle will suffice to keep ether or chloroform out of the eyes, and the use of a Schimmelbusch mask in preference to Skinner's pattern makes any burn of the face very unlikely. It is obvious that a drop or two of either anæsthetic running over the cheek will not burn, otherwise those prepared in the usual way for operation would suffer severely and anæsthetists would seldom be able-bodied. To burn a face in this way requires prolonged contact, which can be achieved only by gross carelessness on the administrator's part. Nevertheless, if in any given patient a facial peculiarity, such as a prominent nose or a nutcracker jaw, points to the possibility of contact with the lint of the mask, then the use of vaseline is highly desirable. A small pot of this should consequently always form part of the anæsthetist's outfit.

### A New Ether Inhaler.

In giving ether by the open method on a gauze or lint mask, the anæsthetic has to be dropped on so liberally that there is always the risk of causing the face burns to which allusion has just been made. The usual plan of obviating this is to pack dry gauze plentifully all round the line of contact of the mask with the face. A new face-piece and inhaler, known as the Roth, or Roth-Dræge, apparatus is now on the market which avoids any risk of burns and for which other advantages are claimed. It consists of a metal facepiece moulded in a different way to any so far introduced into Britain. Unquestionably this fits the ordinary face much better than any other rigid type: it is quite wonderful what close apposition can be obtained with the minimum of pressure. The top of the face-piece is pierced by a valve for inspiration, the side by one for expiration. These valves are similar to those of the Vernon Harcourt apparatus: at least they close with a sharp clicking just as do the latter, so that the anæsthetist knows at once if his patient is not breathing, or is getting air through imperfect adjustment of the face-piece.

A dome fits on to the face-piece in which gauze is packed: ether is then poured into it. The whole apparatus is constructed of plated metal; thus it can be properly sterilised by boiling, a very great advantage. Mr. J. S. Ross recommends* that induction should be by the usual gas-ether sequence, as by this means the patient is more quickly and pleasantly "put under" than by open ether, and is spared a long period of inhalation of strong and icy-cold ether vapour. He favours its use especially for short-necked alcoholic men, goitre cases, and other severe neck operations: these are, of course, just those which so often give trouble with the usual methods; any method of dealing satisfactorily with them is to be welcomed. It is said that many administrators in the north of England are adopting this inhaler.

* *Edinburgh Medical Journal*, July 1909, p. 35.

